# Diagnostic value of various liquid biopsy methods for pancreatic cancer: A systematic review and meta-analysis

**DOI:** 10.1097/MD.0000000000018581

**Published:** 2020-01-17

**Authors:** Yuzhou Zhu, Hao Zhang, Nan Chen, Jianqi Hao, Hongyu Jin, Xuelei Ma

**Affiliations:** a Department of Biotherapy, Cancer Center; b Department of Gastrointestinal Surgery; c Department of Pancreatic Surgery, West China Hospital, Sichuan University; d West China School of Medicine; e State Key Laboratory of Biotherapy and Cancer Center, West China Hospital, Sichuan University, Chengdu, Sichuan Province, China.

**Keywords:** CTCs, ctDNA, diagnostic value, exosomes, liquid biopsy, meta-analysis, pancreatic cancer

## Abstract

**Background:**

Liquid biopsy is a novel method for cancer diagnosis, which has been applied in lung and breast cancers, demonstrating high diagnostic value. However, clinical value of it in pancreatic cancer (PC) remains to be verified. The aim of this meta-analysis was to evaluate overall diagnostic value of various liquid biopsy methods (circulating tumor DNA, circulating tumor cells and exosomes) in detecting PC.

**Methods:**

We comprehensively searched relevant studies in PubMed, Medline, Embase, and Web of Science without time limitation according to PRISMA. Data necessary for reconstructing a 2 × 2 table was calculated from the original articles. The methodological quality of included studies was evaluated by QUADAS-2. Statistical analysis including was performed by the software Meta-Disc version 1.4, and STATA 14.2.

**Results:**

A total of 19 studies including 1872 individuals were included in this meta-analysis. In which, 7 were studies about ctDNA, 7 were on CTCs and 6 were about exosomes (Sefrioui D^1^, studied diagnostic accuracy of both ctDNA and CTCs, with no common patients in these 2 groups). The pooled sensitivity estimates for ctDNA, CTCs and exosomes in detecting PC with their 95% confidential intervals (95% CI) were 0.64 (95%CI 0.58–0.70), 0.74 (95%CI 0.68–0.79) and 0.93 (95%CI 0.90–0.95), respectively. The pooled specificity estimates were 0.92(95%CI 0.88–0.95), 0.83 (95%CI 0.78–0.88) and 0.92 (95%CI 0.88–0.95), respectively. The area under curve (AUC) of the sROC for ctDNA, CTCs and exosomes in detecting PC were 0.9478, 0.8166, and 0.9819, respectively. The overall sensitivity, specificity and AUC of the sROC curve for overall liquid biopsy in detecting PC were 0.80 (95%CI 0.77–0.82), 0.89 (95%CI 0.87–0.91) and 0.9478, respectively.

**Conclusion:**

This meta-analysis confirmed that liquid biopsy had high diagnostic value in detecting PC. In ctDNA, CTCs and exosomes these 3 subgroups, exosomes showed highest sensitivity and specificity.

## 1 Introduction

Pancreatic cancer (PC) is a bleak disease and the fourth leading reason of cancer-associated death in Western countries, causing 41,000 deaths in the US in the year 2016 by estimation. In the past 25 years, the prognosis of PC remains unimproved. The overall survival of 5-year is 8% and the median survival is still shorter than 6 months.^[2]^ Even in patients undergoing adjuvant chemotherapy and resection for PC, prognosis is still poor with a median overall survival of about only two years.^[3]^ This situation is mainly owing to the late diagnosis of PC. Surgical resection is the only radical treatment, but at the time of diagnosis, only a few patients (10%–15%) have localized disease. Consequently, early diagnosis and radical surgery is necessary to improve the prognosis for patients with PC.^[4]^

Clinically, PC is usually diagnosed by biochemical examination, imaging examination and tissue biopsy. However, the specificity of biochemical examination is low: Carbohydrate antigen 19–9 is the only widely used biochemical examination method for PC detection, nevertheless, the area under curve is 0.7 when diagnosing patients with PC from healthy controls.^[5]^ Imaging examination is inefficient to detect early lesions or to differentiate benign from malignant lesions.^[6]^ Tissue biopsy is invasive, and it shows low sensitivity which requires repeated sampling.^[7,8]^ Liquid biopsy, on the other hand, has been proved as a more accurate tool for PC diagnosis.^[9]^ Liquid biopsy is performed by analyzing and sampling of non-solid biological tissues, mainly ctDNA (DNA released into the plasma by tumor cells, as a result of cell death.^[10]^), circulating tumor cells (CTCs) (cells shed from primary cancer into the lymphatics or vasculature and are carried throughout the body.^[11]^) and exosomes (micro-vesicles secreted by cancer or normal cells, with diameter of 30–100 nm.^[12]^) in the blood as test samples.

In the last decade, liquid biopsy has exhibited great diagnostic value of cancers in lung, brain and breast.^[11,13]^ Studies applying circulating tumor DNA (ctDNA), CTCs and exosomes into the diagnosis of PC have also been initiated. Nevertheless, some of these studies included insufficient number of patients, and the results in various articles varied considerably,^[14–16]^ resulting in incomplete and inaccurate conclusions. Moreover, diagnostic value of liquid biopsy for PC was evaluated in subtype of ctDNA, CTCs and exosomes separately, never combined or get comprehensive comparison. Herein, we evaluated the diagnostic value of ctDNA, CTCs, exosomes and overall liquid biopsy for PC, and to compare diagnostic the performance of ctDNA, CTCs, and exosomes.

## 2 Methods

### 2.1 Literature search strategy

Ethical approval was not required, because this literature was a meta-analysis. We searched relevant articles in Embase, PubMed, Medline, and Web of Science database up to August 2019 without date restrictions. The search strategy included (liquid biopsy OR fluid biopsy OR circulating tumor cell OR CTC OR cell-free DNA OR cfDNA OR ctDNA OR circulating tumor DNA OR exosome) AND (Sensitivity OR diagnosis OR diagnostic) AND (Pancreatic Neoplasm OR Pancreas Neoplasms OR Cancer of Pancreas OR Pancreas Cancers OR Pancreas Cancer OR Pancreatic Cancer OR Pancreatic Cancers)To expand search results, we reviewed all references in included articles.

### 2.2 Inclusion and exclusion criteria

Two reviewers evaluated potential articles independently, according to the inclusion and exclusion criteria mentioned below. Afterwards, a blind cross-check was performed to check potential differences. If there were any inconsistencies, a third reviewer was assigned to adjudicate the conflict. The recognition, inclusion, and exclusion of articles were conducted in accordance with preferred reporting items for systematic reviews and meta-analysis (PRISMA) guidelines.

Inclusion criteria were as follows:

(1) human-based studies.
(2) 2)studies sample size included at least 20 individuals;
(3) absolute numbers of true-positive (TP), false-positive (FP), false-negative (FN) and true-negative (TN) were presented in a 2 × 2 contingency table or could be calculated from the study.

The following researches were excluded:

(1) animal studies
(2) insufficient data to construct a 2 × 2 contingency table
(3) case reports, meta-analysis, reviews, or comments.

### 2.3 Data extraction and quality assessment

Two researchers reviewed all eligible papers and extracted the data on a standardized form independently. Data extracted from studies included author, year of publication, country, number of enrolled patients, characteristics of control group, markers for ctDNA, CTCs, exosomes, and absolute numbers of true positive (TP), true negative (TN), false positive (FP) and false negative (FN). If more than one set of data (TP, FP, FN and TN) could be calculated from 1 study, the set of data (TP, FP, FN and TN) with best diagnostic performance was chosen.

The quality of each literature included in this meta-analysis was assessed with the QUADAS-2 checklist.

### 2.4 Statistical analysis

2 × 2 tables were adopted to sort the data, including the absolute numbers of the TP, false-positive, false-negative and true-negative on per-patient basis. These data were combined quantitatively to present the diagnostic performance of ctDNA, CTCs, exosomes, and overall liquid biopsy, separately. We calculated the pooled sensitivities and 95%CI, pooled specificities and 95%CI, pooled positive likelihood ratios (PLR) and 95% CI, pooled negative likelihood ratios (NLR) and 95% CI for ctDNA, CTCs, exosomes and overall liquid biopsy. We also calculated the areas under summary curves for ctDNA, CTCs, exosomes and overall liquid biopsy, respectively. The heterogeneity of the included studies was assessed by both threshold effect and non-threshold effect. If the *P* value of the Spearman correlation coefficient was less than .05, a threshold effect would be identified. If *I*^2^ of Higgin *I*^2^ test was greater 50%, a non-threshold effect would exist. A random effects model was performed as the statistical heterogeneity in the group of ctDNA, CTCs, exosomes and overall liquid biopsy. Publication bias was assessed by Deeks funnel test. *P* value of Deeks funnel test less than .05 indicated significant publication bias. The mentioned approach was carried out by the software Meta-disc version 1.4 (Universidad Complutense, Madrid, Spain) and STATA 14.2 (StataCorp, USA).^[17–19]^

### 2.5 Subgroup analysis

Subgroup analysis on technical differences of ctDNA, CTCs and exosomes were performed. For CTCs, we compared the types of detection methods (CTC-chip, Cell-search, Magnetic beads, Screen-cell and GEDI). For exosomes, we compared the types of detection methods (PCR vs flow cytometry). For ctDNA, PCR was the only methods for detection. If one subgroup contained only 2 articles, we did not calculate the AUC. If one subgroup contained only 1 article, we just listed the indexes from the original article.

## 3 Results

### 3.1 Search results

**Figure 1:**
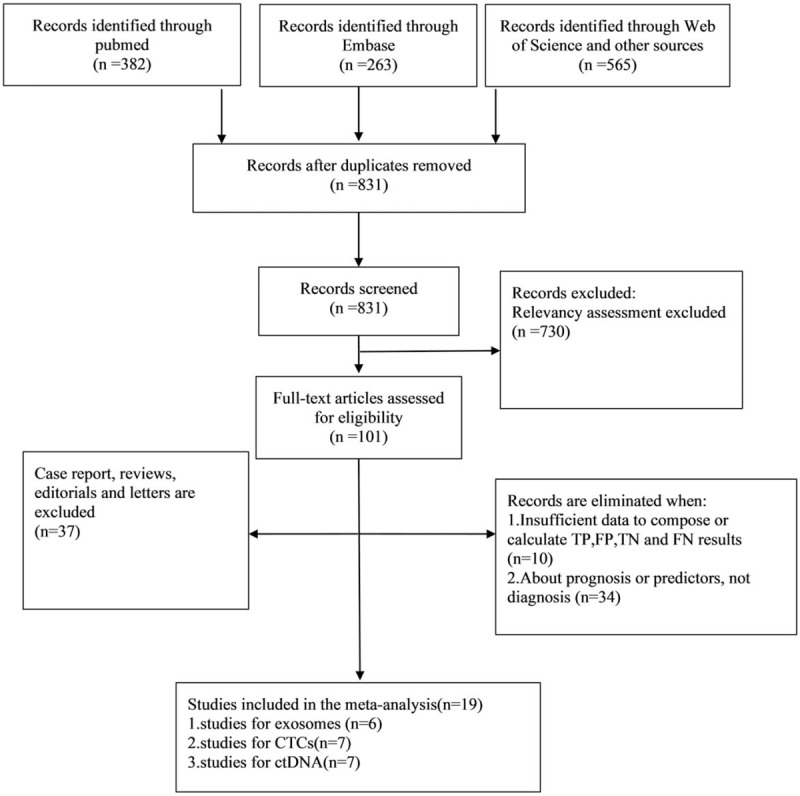
PRISMA flow chart of the literature selection process.

Computerized search generated 1210 articles, after removal of duplicates, reading the abstracts, titles and full-text, 19 articles including 1872 individuals were finally included.^[1,14–16,20–34]^ Among them, 7 studies reported the performance of ctDNA,^[1,14,15,20–23]^ 7 for CTCs,^[1,24–29]^ and 6 for exosomes.^[16,30–34]^ (Sefrioui D^1^, studied diagnostic accuracy of both ctDNA and CTCs, with no common patients in these two groups). Since cfDNA (Circulating cell-free DNA) were not distinguished from ctDNA in many studies, these 2 were put together in this meta-analysis. Figure 1 shows the flow chart of the literature selection process.

### 3.2 Summary of included studies

For exosomes detection, PCR and flow cytometry were the 2 methods adopted by the included articles. For ctDNA detection, PCR was the only method adopted by the included articles. For CTC detection, CTC-chip, magnetic beads, screen-cell, cell-search and GEDI were all methods adopted by included articles.

Table 1 presents detailed characteristics of the 19 included articles, including author, year of publication, country, number of enrolled patients, characteristics of control group, markers for ctDNA, CTCs, exosomes, and absolute numbers of true positive, true negative, false positive, and false negative. If different cut-off points were adopted in one study, we chose the one generating best balance of sensitivity and specificity.

**Table 1:**
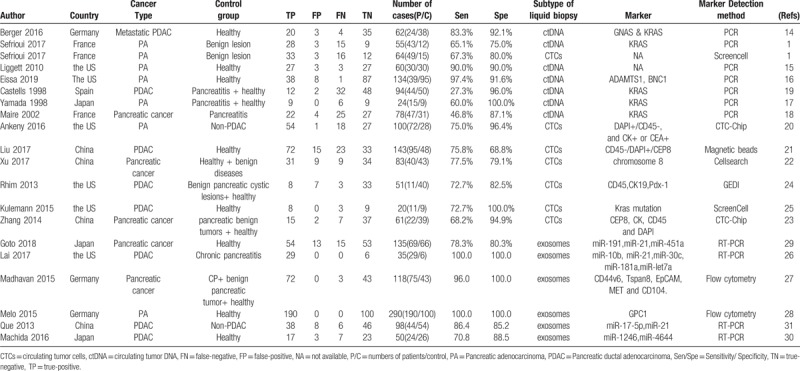
Characteristics of 19 studies included in the meta-analysis.

### 3.3 Quality of the included studies

**Figure 2:**
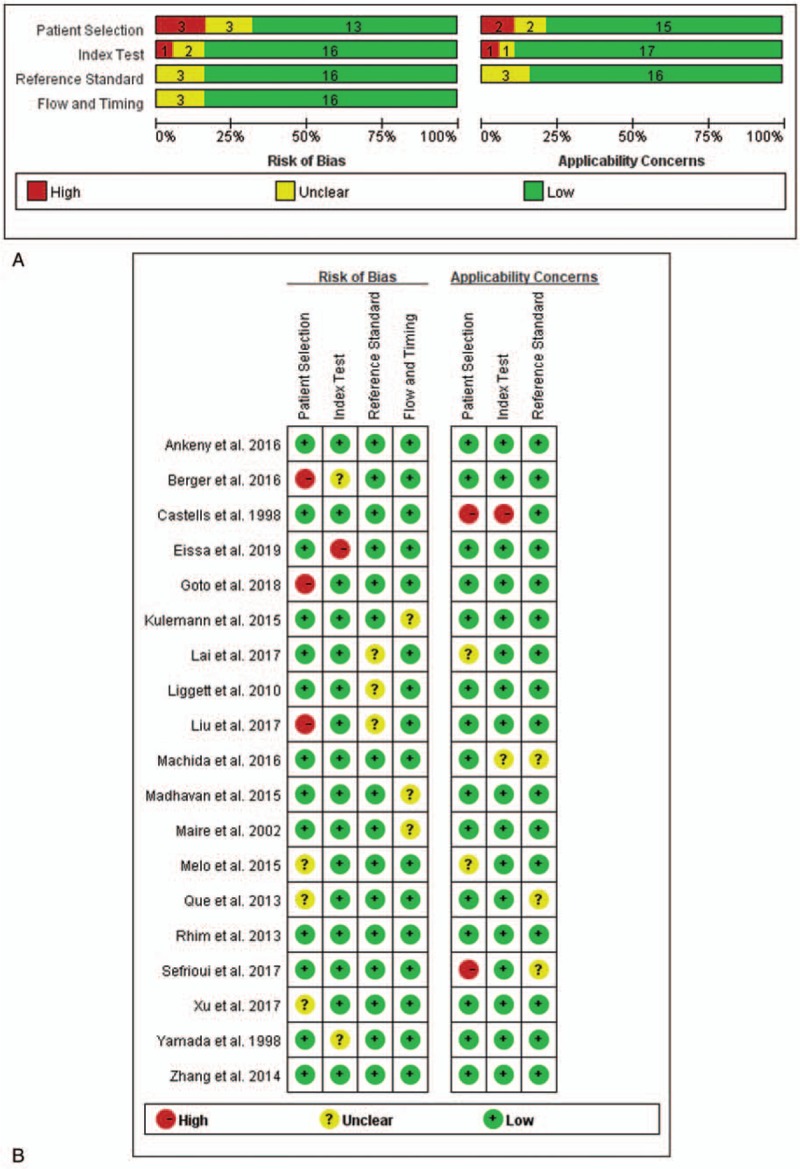
Quality assessment of studies by Quadas-2 evaluation tool. (A) Risk of bias graph. (B) Risk of summary.

Standard quality evaluation of the 19 included studies was conducted on the basis of Quadas-2^[35,36]^ tool. Quality valuation were performed and shown in Figure 2. According to this evaluating system, the 19 included studies were finally defined as reliable, with the quality ranging from moderate to high.

### 3.4 Diagnostic performance for overall liquid biopsy

All pooled results were shown in Table 2. After pooling 19 studies, the sensitivity, specificity, PLR, NLR, DOR, and AUC of overall liquid biopsy in detecting PC, was 0.80 (95%CI 0.77–0.82), 0.89 (95%CI 0.87–0.91), 6.39 (95%CI 4.29–9.52), 0.24 (95%CI 0.15–0.37), and 31.11 (95%CI 16.10–60.14), respectively. The AUC of sROC was 0.9360, which suggested a strong diagnostic value. Exosomes, CTCs and ctDNA were 3 stratification of liquid biopsy. Forest plot of sensitivity, specificity, PLR, NLR DOR, and sROC of overall liquid biopsy were shown in Figure 3  .

**Table 2:**
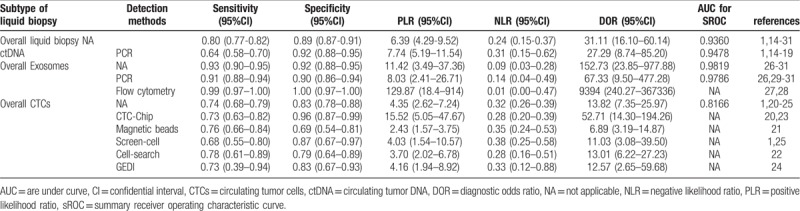
Summary results of subgroup analysis for liquid biopsy in the diagnosis of pancreatic cancer.

**Figure 3:**
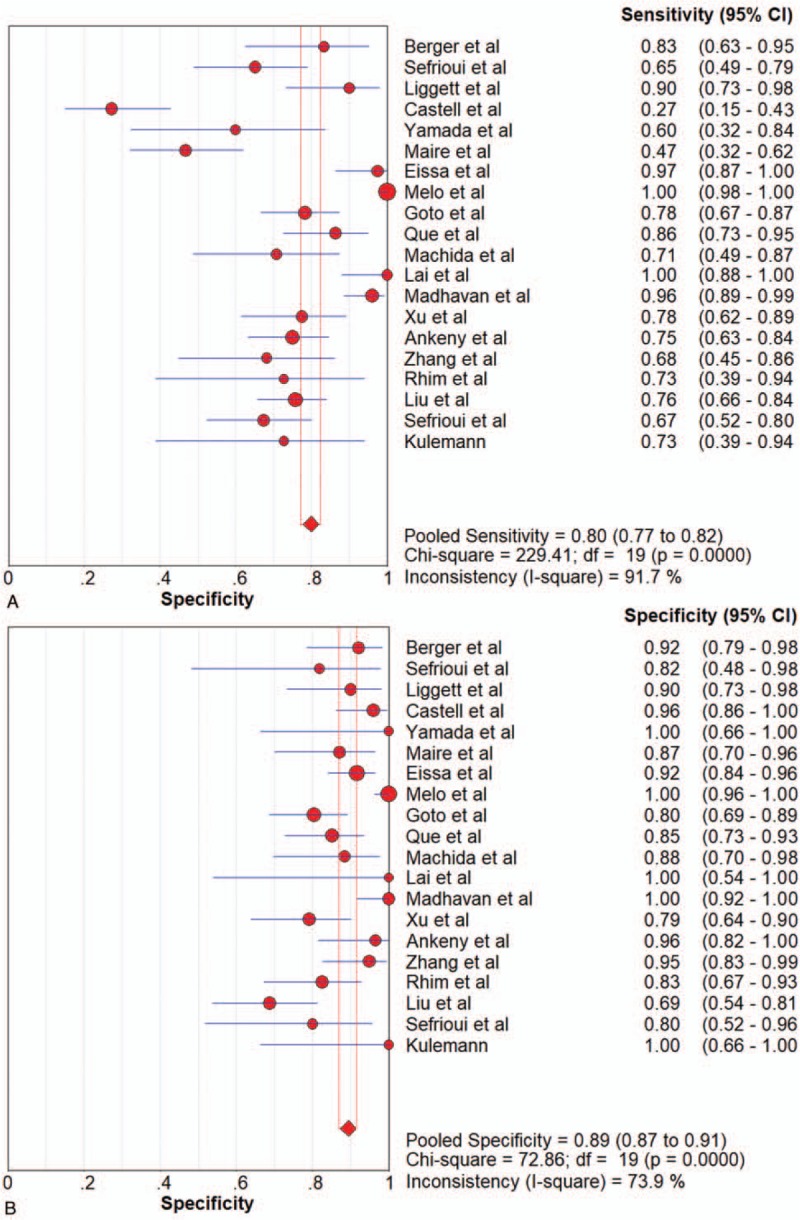
Forest plots of the diagnostic value for overall liquid biopsy in detecting pancreatic cancer. (A) Sensitivity. (B) Specificity. (C) positive likelihood ratio. (D) negative likelihood ratio. (E) Diagnostic odds ratio. (F) SROC curve.

**Figure 3 (Continued):**
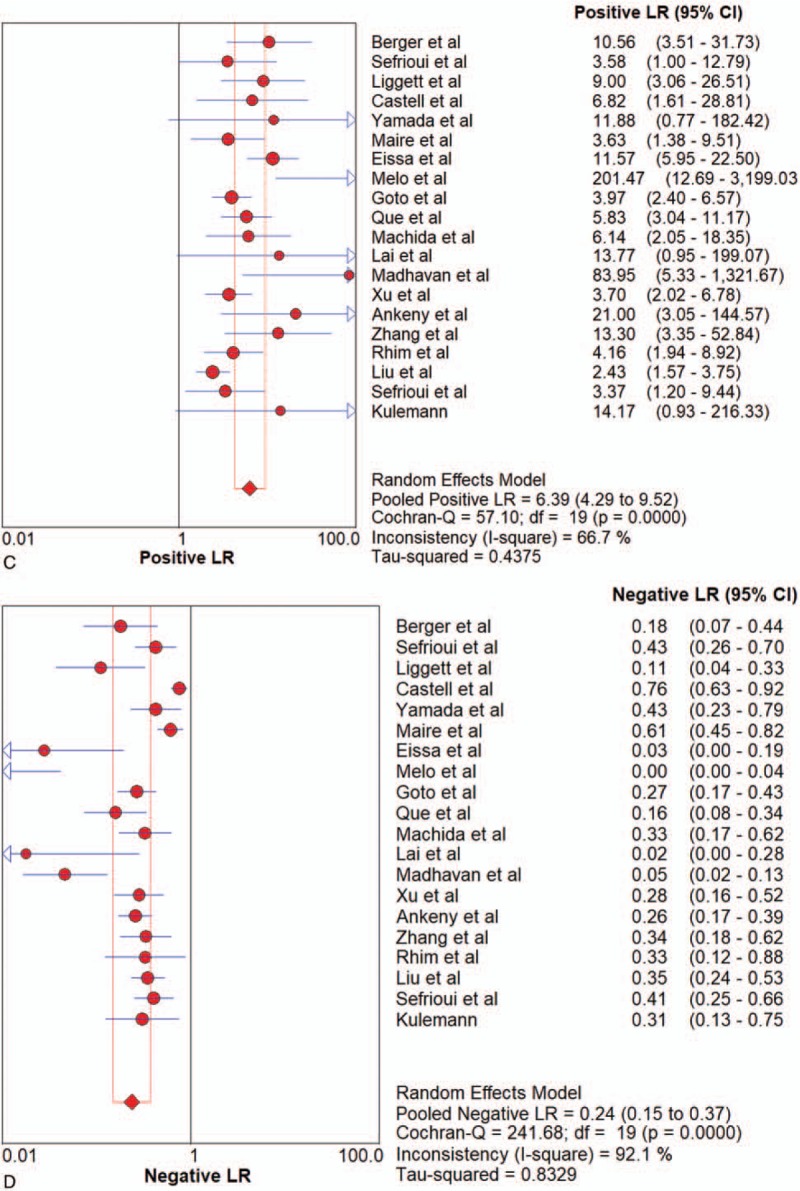
Forest plots of the diagnostic value for overall liquid biopsy in detecting pancreatic cancer. (A) Sensitivity. (B) Specificity. (C) positive likelihood ratio. (D) negative likelihood ratio. (E) Diagnostic odds ratio. (F) SROC curve.

**Figure 3 (Continued):**
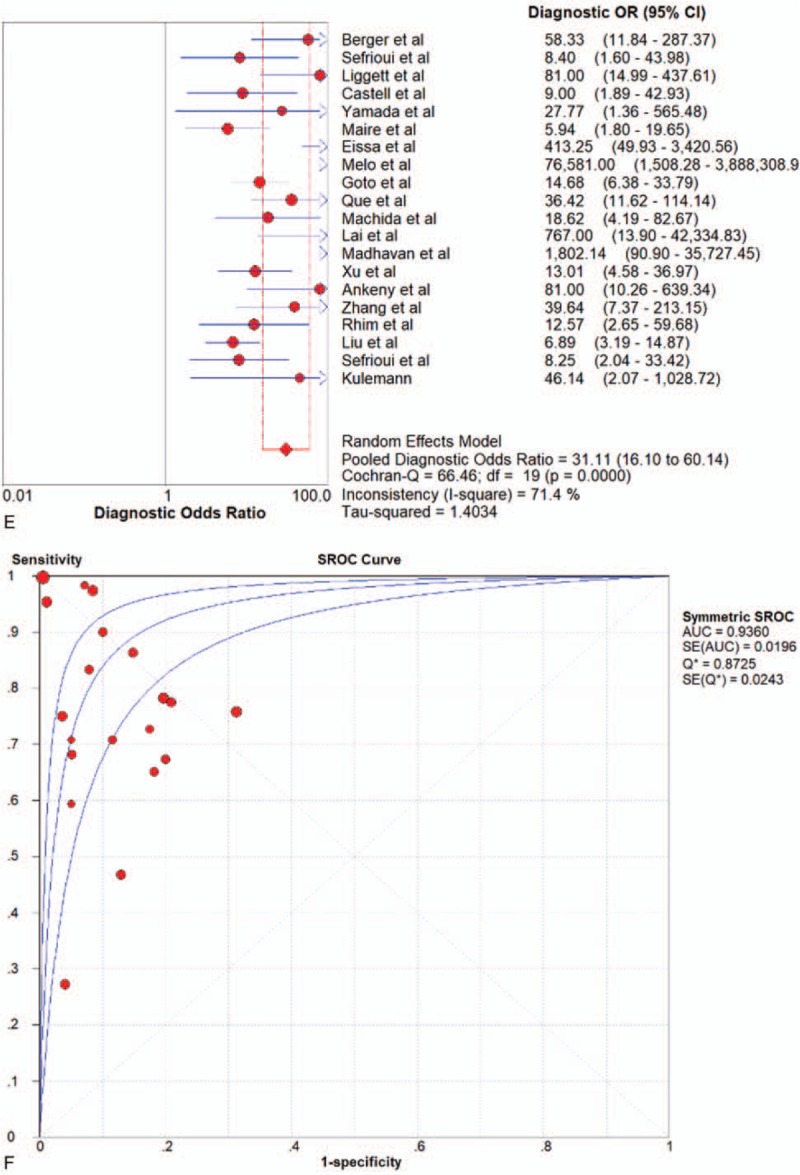
Forest plots of the diagnostic value for overall liquid biopsy in detecting pancreatic cancer. (A) Sensitivity. (B) Specificity. (C) positive likelihood ratio. (D) negative likelihood ratio. (E) Diagnostic odds ratio. (F) SROC curve.

### 3.5 Diagnostic performance for ctDNA

Seven articles were included. PCR was the only method adopted by the included articles All of the 7 studies of ctDNA had a pooled sensitivity, specificity, PLR, NLR, DOR and AUC of 0.64 (95%CI 0.58–0.70), 0.92 (95%CI 0.88–0.95), 7.74 (95%CI 5.19–11.54), 0.31 (95%CI 0.15–0.62), 27.29 (95%CI 8.74–85.20), and 0.9478 (Fig. 4  and Table 2), which suggested a strong diagnostic value.

**Figure 4:**
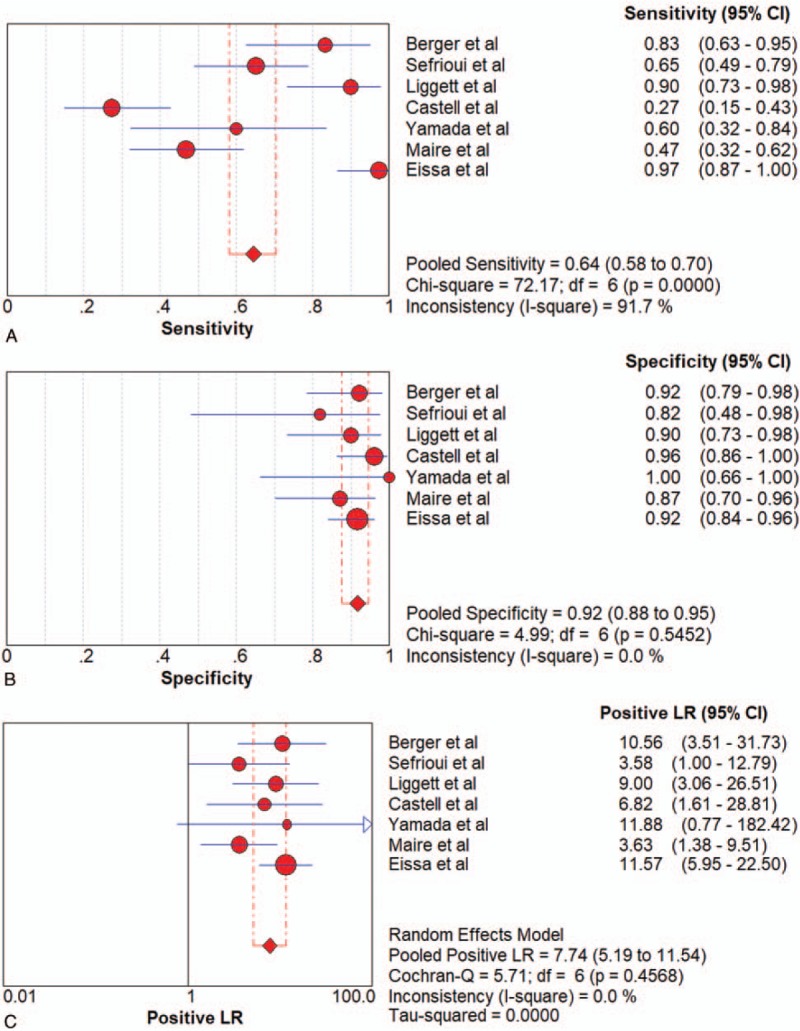
Forest plots of the diagnostic value for ctDNA in detecting pancreatic cancer. (A) Sensitivity. (B) Specificity. (C) positive likelihood ratio. (D) negative likelihood ratio. (E) Diagnostic odds ratio. (F) SROC curve.

**Figure 4 (Continued):**
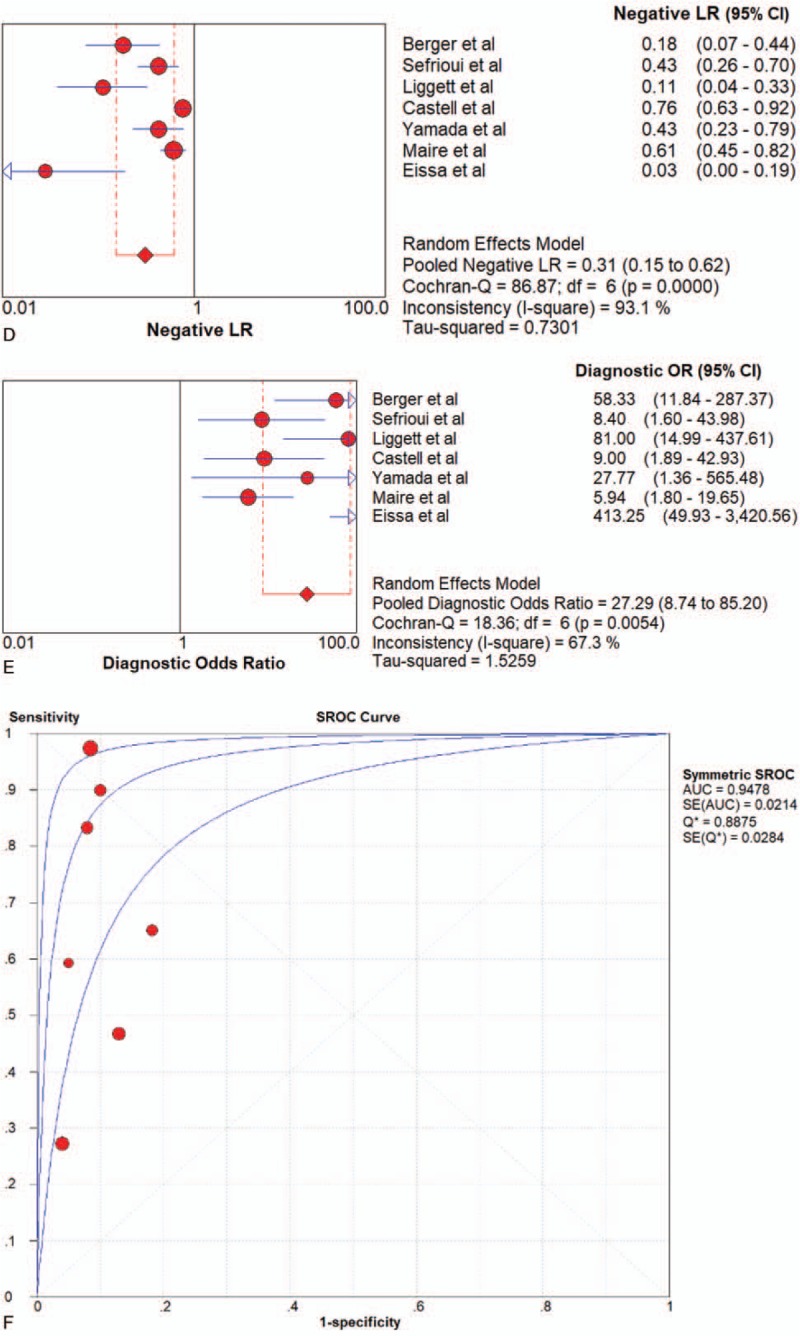
Forest plots of the diagnostic value for ctDNA in detecting pancreatic cancer. (A) Sensitivity. (B) Specificity. (C) positive likelihood ratio. (D) negative likelihood ratio. (E) Diagnostic odds ratio. (F) SROC curve.

### 3.6 Diagnostic performance for exosomes

Six articles were included. PCR and flow cytometry were the 2 methods adopted by the included articles. Pooled sensitivity, specificity, PLR, NLR, DOR and AUC were 0.91 (95%CI 0.88–0.94), 0.90 (95%CI 0.86–0.94), 8.03 (95%CI 2.41–26.71), 0.14 (95%CI 0.04–0.49), 67.33 (95%CI 9.50–477.28) and 0.9786 in PCR group, and 0.99 (95%CI 0.97–1.00), 1.00 (95%CI 0.97–1.00), 129.87 (95%CI 18.44–914.78), 0.01 (95%CI 0.00–0.47) and 9394 (95%CI 240.27–367336.80) in flow cytometry group, respectively. Since there were only 2 studies adopting flow cytometry to detect exosomes, AUC in flow cytometry group was unavailable. All of the 6 studies of exosomes had a pooled sensitivity, specificity, LR+, LR–, DOR and AUC of 0.93 (95%CI 0.90–0.95),0.92 (95%CI 0.88–0.95),11.42 (95%CI 3.49–37.63),0.09 (95%CI 0.03–0.28),152.73 (95%CI 23.85–977.88), and 0.9819, respectively (Fig. 5  and Table 2).

**Figure 5:**
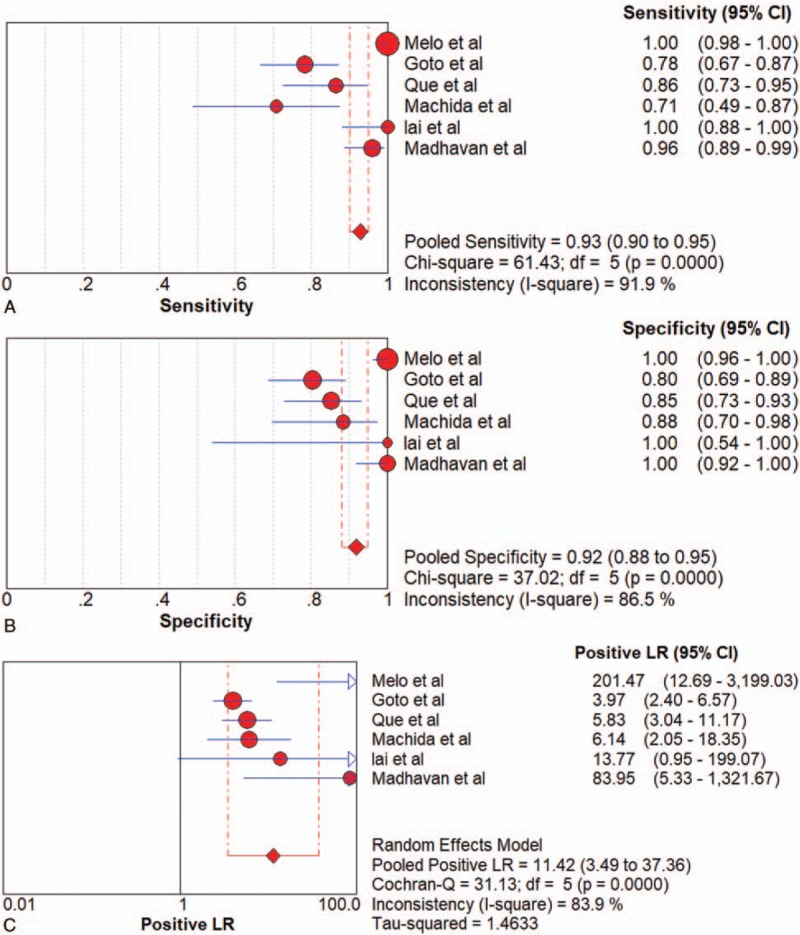
Forest plots of the diagnostic value for overall exosomes in detecting pancreatic cancer. (A) Sensitivity. (B) Specificity. (C) positive likelihood ratio. (D) negative likelihood ratio. (E) Diagnostic odds ratio. (F) SROC curve.

**Figure 5 (Continued):**
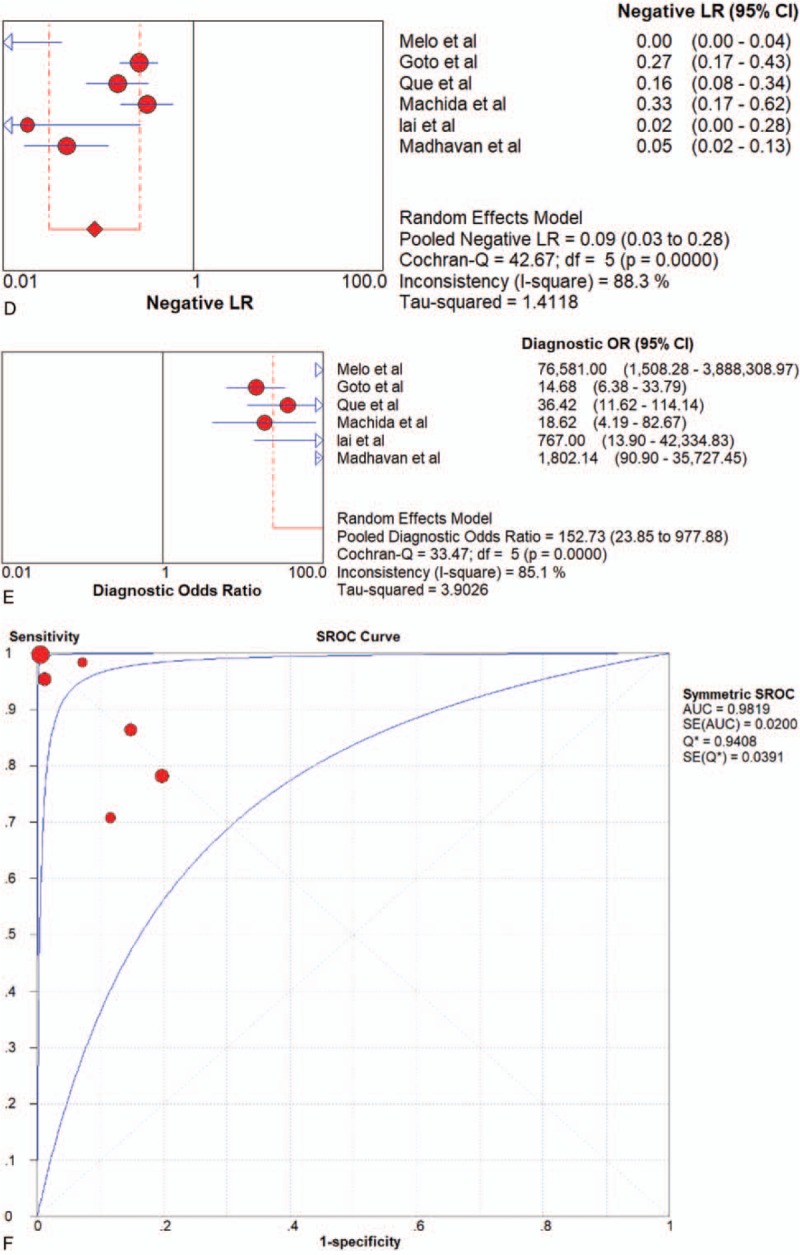
Forest plots of the diagnostic value for overall exosomes in detecting pancreatic cancer. (A) Sensitivity. (B) Specificity. (C) positive likelihood ratio. (D) negative likelihood ratio. (E) Diagnostic odds ratio. (F) SROC curve.

### 3.7 Diagnostic performance for CTCs

Seven articles were included. CTC-chip, magnetic beads, screen-cell, cell-search and GEDI were all methods adopted by included articles. Since there was only 1 article that used magnetic beads, cell-search and GEDI, respectively, the results shown in Table 2 came directly from these studies. Pooled sensitivity, specificity, LR+, LR–, DOR were 0.73 (95%CI 0.63–0.82), 0.96 (95%CI 0.87–0.99), 15.52 (95%CI 5.05–47.67), 0.28 (95%CI 0.20–0.39) and 52.71 (95%CI 14.30–194.26) in CTC-chip group, and 0.68 (95%CI 0.55–0.80), 0.87 (95%CI 0.67–0.97), 4.03 (95%CI 1.54–10.57),0.38 (95%CI 0.25–0.58) and 11.03 (95%CI 3.08–39.50) in Screen-cell group, respectively. All of the 7 studies of CTCs had a pooled sensitivity, specificity, PLR, NLR, DOR and AUC of 0.74 (95%CI 0.68–0.79), 0.83 (95%CI 0.78–0.88), 4.35 (95%CI 2.62–7.24), 0.32 (95%CI 0.26–0.39), 13.82 (95%CI 7.35–25.97) and 0.8166 respectively, (Fig. 6  and Table 2), which suggested a moderate diagnostic value.

**Figure 6:**
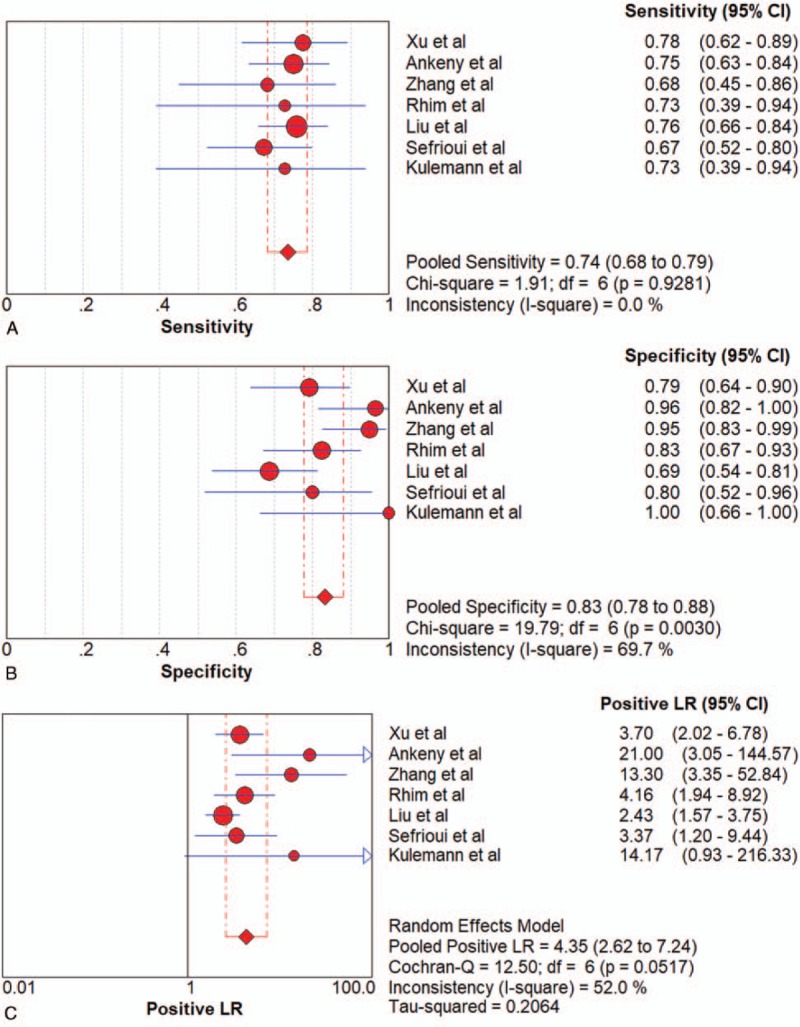
Forest plot of the diagnostic value for overall CTCs in detecting pancreatic cancer. (A) Sensitivity. (B) Specificity. (C) positive likelihood ratio. (D) negative likelihood ratio. (E) Diagnostic odds ratio. (F) SROC curve.

**Figure 6 (Continued):**
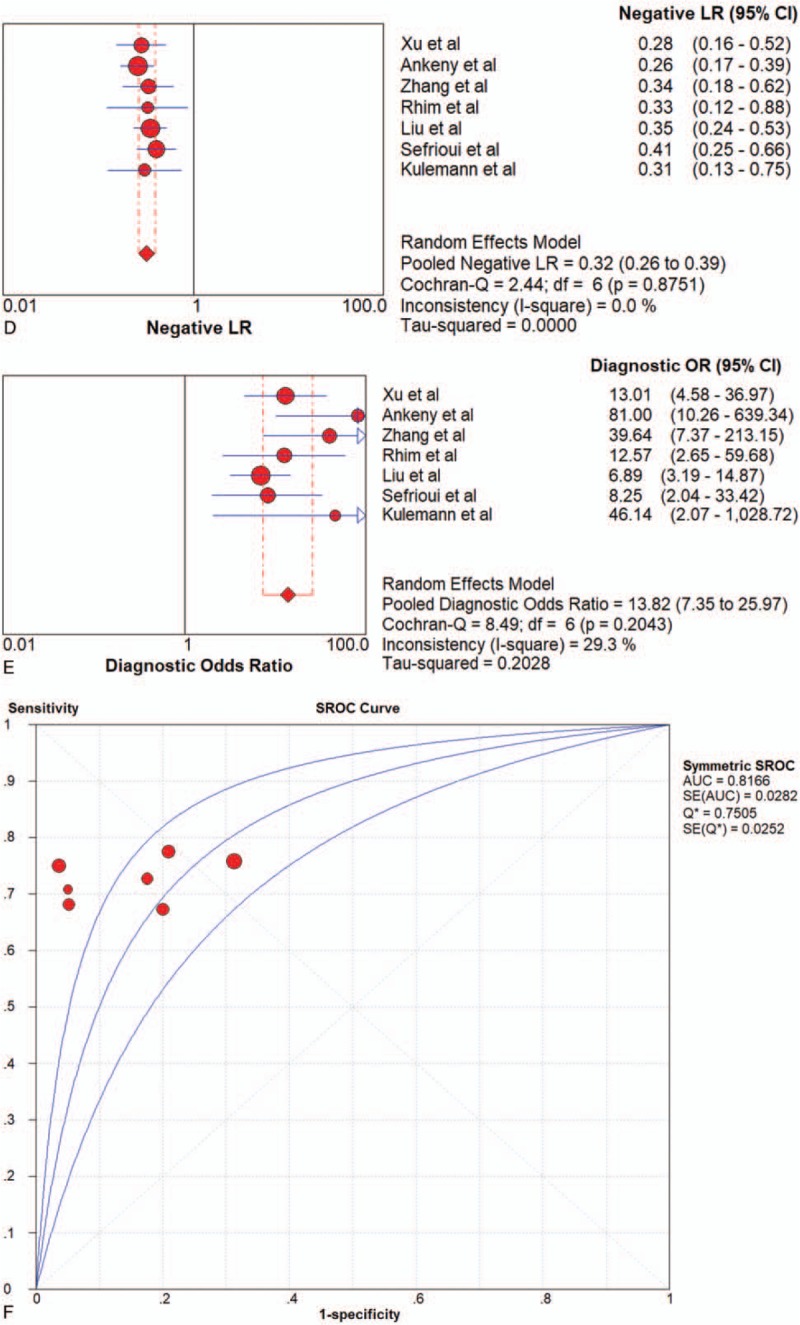
Forest plot of the diagnostic value for overall CTCs in detecting pancreatic cancer. (A) Sensitivity. (B) Specificity. (C) positive likelihood ratio. (D) negative likelihood ratio. (E) Diagnostic odds ratio. (F) SROC curve.

### 3.8 Heterogeneity and publication bias

**Figure 7:**
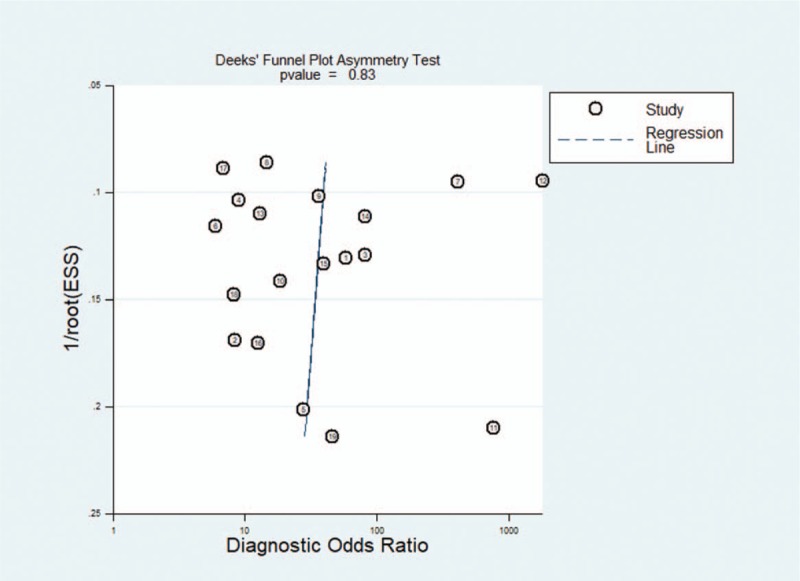
Result of Deeks funnel test.

Significant heterogeneity across the studies was detected according to the *I*^2^ value of DOR (67.3%, 85.1%, and 71.4% for ctDNA, exosomes, and overall liquid biopsy, respectively). While no significant heterogeneity across the studies for CTCs according to the *I*^2^ value of DOR (29.3%). *P* value of Deeks funnel test was .83, suggesting no significant publication bias existed. (Results of Deeks funnel test was shown in Fig. 7)

## 4 Discussion

Previous systematic reviews indicated that some subtype of liquid biopsy could be adopted to detect PC. This meta-analysis pooled the data from 19 studies about the diagnostic performance of liquid biopsy, and the result showed overall liquid biopsy had a sensitivity, specificity of 0.80 (95%CI 0.77–0.82), 0.89 (95%CI 0.87–0.91), respectively. AUC is widely accepted as a strong index for evaluating the overall accuracy of diagnostic tests. If AUC is more than 0.9, the diagnostic efficiency is considered to be powerful.^[30]^ The AUC of overall liquid biopsy in detecting PC was 0.936, which indicated that liquid biopsy was a powerful test for pancreatic detection.

Exosomes are membrane-bound nano-capsules that can transfer a variety of bioactive molecules (such as proteins, lipids and microRNAs) from donor cells to recipient cells.^[37]^ A total of 6 papers that studied the exosomes performance were included. The summary sensitivity, specificity and AUC of exosomes were 0.93 (95%CI 0.90–0.95), 0.92 (95%CI 0.88–0.95), and 0.9819. The AUC of exosomes was 0.9819, which showed exosomes had strong diagnostic value. The following reasons might contribute to high diagnostic value of exosome: For sensitivity, pancreatic cells had strong exocrine function, making exosomes in peripheral blood high and easy to be detected. For specificity, the function of exosome was to transfer a variety of bioactive molecules (such as proteins, lipids and microRNAs) from donor cells to recipient cells. PC cells were active and usually require some special signaling molecules. PCR and flow cytometry were the two methods to detect exosome adopted by the included articles. PCR was usually used to detect exosomes containing DNA or RNA with sensitivity and specificity of 0.91 (95%CI 0.8–0.94) and 0.90 (95%CI 0.86–0.94), respectively, while flow cytometry for protein, with sensitivity and specificity of 0.99 (95%CI 0.97–1.00) and 1.00 (95%CI 0.97–1.00).

CTCs are cells shedding from primary and secondary tumors and enter the bloodstream.^[38]^ A total of 7 papers that studied CTCs performance were included. The summary sensitivity, specificity and AUC of CTCs were 0.74 (95%CI 0.68–0.79), 0.83 (95%CI 0.78–0.88), and 0.8166. Compared with exosome, the sensitivity of CTCs was lower, which could result from three reasons:

(1) CTCs were easier to be trapped in the liver when blood flew through the portal vein and entered the systemic circulation.^[39]^
(2) Compared with normal pancreatic tissues, the blood flow of pancreatic malignant tumors was reduced by 60%, so the chances of tumor cells invading the blood flow were less.^[40]^

Compared with exosome, the specificity of CTCs was also lower. Although the sensitivity of CTCs was lower than exosome, the AUC indicated that CTCs still had strong diagnostic value in detecting PC. CTC-Chip, Magnetic beads, Screen-cell, Cell-search and GEDI were methods to detect CTCs adopted by the included articles. Because the number of articles included in each subgroup was not enough, we only listed sensitivity, specificity, PLR and NLR of each subgroup. The result indicated that CTC-chip group had significant higher specificity, while sensitivity between each group was not significantly different.

Circulating tumor DNA is generally believed to be released by necrotic or apoptotic cells in CTCs, primary tumors or secondary deposit tumors.^[9,32,41]^ A total of 7 papers that studied ctDNA performance were included. The summary sensitivity, specificity and AUC of ctDNA were 0.64 (95%CI 0.58–0.70), 0.92 (95%CI 0.88–0.95), and 0.9478. The sensitivity of ctDNA was slightly lower than CTCs, which had been supported by some studies.^[42]^ This may be partly due to the low content of ctDNA in peripheral blood. Moreover, for the early stage of cancer, the amount of ctDNA was probably only 1 genome per 5 ml of plasma.^[43]^ Therefore, effectively capturing ctDNA was still technically challenging. The specificity of ctDNA was much higher than CTCs. The reason may be

(1) ctDNA usually could be detected after necrosis of a large number of tumor cells, when the tumor burden is already very high.
(2) Gene mutations in PC were usually specific.

Although the sensitivity of ctDNA was low, the specificity and AUC of ctDNA was high.

According to comprehensive comparation of ctDNA, CTCs, exosomes and overall liquid biopsy, we found that liquid biopsy had strong diagnostic performance in detecting PC. Among the subgroup detection methods, exosomes had highest overall diagnostic value, sensitivity and AUC. It showed that exosomes were suitable for the diagnosis and screening of PC. The ctDNA group had the highest specificity 0.92 (95CI% 0.88–0.95), the same as exosome group. However, ctDNA group had the lowest sensitivity. This indicated that ctDNA was suitable for the diagnosis but not screening of PC. CTCs had a medium sensitivity, while lowest specificity and AUC in these 3 groups.

To the best of my knowledge, this meta-analysis was the first and most comprehensive one for the diagnostic value of ctDNA, CTCs, exosomes and overall liquid biopsy for detecting PC. Nevertheless, it still had several limitations requiring attention. At study and outcome level, there were different methods to detect CTCs. However, for each subgroup, the number of included articles was not enough to calculate AUC. Therefore, we only listed the sensitivity, specificity, PLR and NLR of each subgroup. At review-level, relevant studies that have not been published online may be lost. Thirdly, we only extracted data from English and Chinese studies, which may also affect our results.

## 5 Conclusion

After comprehensive analysis of diagnostic value of ctDNA, CTCs, exosomes and overall liquid biopsy for detecting PC, we found that liquid biopsy had great diagnostic value, which was valuable to clinical practice. In these 3 subtypes of liquid biopsy, exosomes were the best one and was suitable for both screening and diagnosis of PC, while ctDNA was suitable for diagnosis only. Although CTCs had lowest AUC and specificity, the AUC was still more than 0.80.

## Author contributions

Yuzhou Zhu developed the search strategy, analyzed the data and wrote the manuscript. Hao Zhang checked the search, and review the manuscript. Jianqi Hao performed data search. Hongyu Jin did the quality assessment. Xuelei Ma reviewed the manuscript and finally approved the version to be published.
